# Class-Specific Histone Deacetylase Inhibitors Promote 11-Beta Hydroxysteroid Dehydrogenase Type 2 Expression in JEG-3 Cells

**DOI:** 10.1155/2017/6169310

**Published:** 2017-02-21

**Authors:** Katie L. Togher, Louise C. Kenny, Gerard W. O'Keeffe

**Affiliations:** ^1^Department of Obstetrics and Gynaecology, Cork University Maternity Hospital, University College Cork, Cork, Ireland; ^2^APC Microbiome Institute, Biosciences Institute, University College Cork, Cork, Ireland; ^3^INFANT Centre, Cork University Maternity Hospital, University College Cork, Cork, Ireland; ^4^Department of Anatomy and Neuroscience, University College Cork, Cork, Ireland

## Abstract

Exposure to maternal cortisol plays a crucial role in fetal organogenesis. However, fetal overexposure to cortisol has been linked to a range of short- and long-term adverse outcomes. Normally, this is prevented by the expression of an enzyme in the placenta called 11-beta hydroxysteroid dehydrogenase type 2 (11*β*-HSD2) which converts active cortisol to its inactive metabolite cortisone. Placental 11*β*-HSD2 is known to be reduced in a number of adverse pregnancy complications, possibly through an epigenetic mechanism. As a result, a number of pan-HDAC inhibitors have been examined for their ability to promote 11*β*-HSD2 expression. However, it is not known if the effects of pan-HDAC inhibition are a general phenomenon or if the effects are dependent upon a specific class of HDACs. Here, we examined the ability of pan- and class-specific HDAC inhibitors to regulate 11*β*-HSD2 expression in JEG3 cells. We find that pan-, class I, or class IIa HDAC inhibition promoted 11*β*-HSD2 expression and prevented cortisol or interleukin-1*β*-induced decrease in its expression. These results demonstrate that targeting a specific class of HDACs can promote 11*β*-HSD2 expression in JEG3 cells. This adds to the growing body of evidence suggesting that HDACs may be crucial in maintaining normal fetal development.

## 1. Introduction

The glucocorticoid hypothesis proposes that overexposure of the fetus to glucocorticoids may produce long lasting effects on fetal development that subsequently increase disease risk later in life [1]. The glucocorticoid hypothesis is affirmed by studies that have shown that elevated maternal cortisol is associated with heightened HPA activity [2] and alterations in brain structure [3] in affected offspring. At the core of this process is the placental enzyme 11*β*-hydroxysteroid dehydrogenase type 2 (11*β*-HSD2), an enzyme that is expressed primarily within the syncytiotrophoblast of the placenta where it catalyses the conversion of active cortisol into its inactive product cortisone, thereby controlling the levels of cortisol that reach the fetus [4]. A number of preclinical and clinical studies have demonstrated a reduction in the placental expression of 11*β*-HSD2 following exposure to prenatal stress [5], anxiety [6], and following maternal infection [7]. In addition to this, placental* HSD11B2* mRNA levels are reduced in pregnancy complications such as preeclampsia [8], intrauterine growth restriction (IUGR) [9], preterm birth (PTB) [10], and low birth weight (LBW) [11].

A complex repertoire of molecular pathways have been shown to be involved in regulating placental* HSD11B2* expression. Inhibition of the mitogen-activated protein kinases (MAPK) ERK1/2 increases* HSD11B2* expression [12], whilst suppressing p38 reduces 11*β*-HSD2 activity [13].* HSD11B2* is increased by activation of peroxisome proliferator-activated receptor delta (PPAR*δ*) [14] through recruitment of the SP1 transcription factor (TF) [15]. Similarly, activation of the hedgehog signalling [16] and forskolin-induced activation of the cyclic AMP (cAMP) pathway increases* HSD11B2* expression [17]. More recently, epigenetic mechanisms have been linked to 11*β*-HSD2 regulation. The most widely studied epigenetic mechanisms are DNA methylation and histone acetylation. Histone acetylation is regulated by histone acetyl transferase (HATs) and histone deacetylase (HDACs) enzymes. HATs add acetyl groups onto the N-terminal tail of histone proteins which increases gene expression [18]. HDACs remove them, thereby repressing transcription [19]. In humans, 18 HDACs have been discovered and they are classed into four main families: class I (HDACs 1, 2, 3, and 8), class II (HDACs 4, 5, 6, 7, 9, and 10), class III (SIRT1, SIRT2, SIRT3, SIRT4, SIRT5, SIRT6, and SIRT7), and class IV (HDAC 11) [20].

Recently, a significant emphasis has been placed on in vitro studies to tease apart the precise epigenetic mechanisms involved in regulating placental 11*β*-HSD2 expression. Global knock down of DNA methylation using the demethylating agent 5-aza-2′-deoxycytidine (5-aza) in JEG-3 cells has been shown to increase the expression of a number steroidogenic genes including* HSD11B2*, indicating a direct link for regulation of* HSD11B2* expression by methylation [21]. Despite advancements being made in understanding the role of methylation in 11*β*-HSD2 expression, little focus has been placed on examining the role that HDACs play in regulating 11*β*-HSD2. The present study aimed to investigate the role of histone acetylation in regulating basal and stressor-induced changes in 11*β*-HSD2 protein expression in an in vitro placenta model using small molecule pharmacological inhibitors.

## 2. Methods

### 2.1. Cell Culture and Treatment

JEG-3 cells were grown in Dulbecco's modified Eagle's medium (DMEM):  F12 (Sigma), with 10% fetal calf serum (FCS), 100 nM L-Glutamine, 100 U/ml penicillin, and 10 *μ*g/ml streptomycin (Sigma). Cells were maintained at 37°C in a humidified atmosphere of 5% CO_2_. 50,000 cells per well were plated on a 24-well plate and were treated with 1, 5, or 10 *μ*M of MC1568, MS275, or SAHA (Selleckchem). Where indicated, 10 ng/ml interleukin-1*β* (IL-1*β*; Promokine) or 2 *μ*M cortisol (Cort; Santa Cruz) was added for 24 h before HDAC inhibitor (HDI).

### 2.2. MTT Assay

To assess cell viability, a thiazolyl blue tetrazolium bromide (MTT) solution was added to the cells at a concentration of 1 mg/ml in HBSS (Sigma). Following a 2-hour (h) incubation at 37°C, the cells were lysed in DMSO (Sigma). Absorbance was measured at a wavelength of 540 nm with a reference wavelength of 630 nm.

### 2.3. Immunocytochemistry

At the experimental end point, cultures were fixed in 4% paraformaldehyde (PFA) in PBS for 10 min. Following 3 × 5 min washes in 10 mM PBS containing 0.02% Triton X-100 (PBS-T), cultures were incubated in blocking solution (5% BSA in PBS-T) for 1 h at room temperature. Where indicated, cultures were incubated in the following primary antibodies: 11*β*-HSD2 (1 : 250; Santa Cruz), AcH3 (1 : 250; Santa Cruz), GR (1 : 250; Santa Cruz), or IL1R1 (1 : 250; Invitrogen) diluted in 1% BSA in 10 mM PBS at 4°C for 16 h. Following 3 × 5 min washes in PBS-T, cells were incubated in the appropriate Alexa Fluor 488-conjugated or 594-conjugated secondary antibodies (1 : 1000; Invitrogen) diluted in 1% BSA in 10 mM PBS at room temperature for 2 h. Cultures were counterstained with DAPI (1 : 3000; Sigma). Cells were imaged under an Olympus IX70 inverted microscope with Olympus DP70 camera and AnalysisD™ software.

### 2.4. RNA Extraction and Real-Time PCR

RNA was extracted from JEG-3 cells 24 hours after seeding and term human placental tissue using Trizol Reagent (Life Technologies). Placental tissue was homogenised with a pestle and mortar and JEG-3 cells were removed from flasks by scraping and incubated in Trizol for 10 min and RNA extraction proceeded according to the manufacturer's instructions. 500 ng of RNA was reverse-transcribed using a high capacity cDNA Reverse Transcription Kit (Applied Biosystems) in a 20 *μ*l reaction mixture consisting of 2.0 *μ*l 10x RT Buffer, 0.8 *μ*l 25x dNTP mix (100 mM), 2.0 *μ*l 10x RT Random Primers, 1.0 *μ*l Reverse Transcriptase, and 4.2 *μ*l Nuclease-free H_2_O, using the following parameters: 25°C for 10 min; 37°C for 120 min; 85°C for 5 min; 4°C for at least 10 min. The cDNA was stored at −80°C prior to use. For real-time PCR, samples were run in duplicate using TaqMan® Gene Expression Assay (Applied Biosystems) for* HSD11B2* using* 18S* as a reference gene under the following parameters: 50°C for 2 min; 95°C for 10 min; 40 repetitions of 95°C for 15 s; and annealing/elongating at 60°C for 1 min.

### 2.5. Immunohistochemistry

Histological placental sections (6 *μ*M) were incubated in blocking solution (5% bovine serum albumin (BSA)) for 1 h at room temperature. Sections were treated with 10% H_2_O_2_ for 5 min, washed in 10 mM Phosphate Buffered Saline (PBS), and blocked for 1 h in 10% normal goat serum in 10 mM PBS with 0.4% Triton X. Sections were incubated in primary antibody to 11*β*-HSD2 (1 : 250; Santa Cruz) in 1% normal goat serum in 10 mM PBS with 0.4% Triton X overnight at 4°C. Following a 3 × 10 min wash in 10 mM PBS, sections were incubated with a biotinylated secondary antibody (1 : 200; Vector Labs) for 2 h at room temperature. Following another 3 × 10 min wash in 10 mM PBS, sections were incubated in ABC solution (1 : 200; Vector Labs) for 45 min at room temperature followed by immersion in diaminobenzidine substrate/chromogen reagent for 2-3 min at room temperature. Sections were dehydrated, cleared, mounted, and imaged using an Olympus AX70 Provis upright microscope.

### 2.6. Statistical Analysis

For real-time PCR, expression levels were calculated using the 2-delta-Ct threshold method [22]. For immunocytochemistry, the fluorescence intensity of individual cells that were immunopositive for 11*β*-HSD2 or AcH3 was measured by densitometry using Image J analysis software (Rasband, WJ, http://rsb.info.nih.gov/ij/). The relative fluorescence intensity of 11*β*-HSD2 or AcH3 was calculated as the average fluorescence intensity after subtraction of the background noise. Data was analysed using GraphPad Prism v 5 (GraphPad Software Inc., San Diego, California). Where indicated, data was analysed (as per Section 2.3) with unpaired Student's *t*-test or one-way ANOVA with Tukey's post hoc testing. Values of *p* < 0.05 were considered statistically significant.

## 3. Results

### 3.1. Distribution of* HSD11B2* in the Human Placenta and JEG-3 Cells

**Figure 1:**
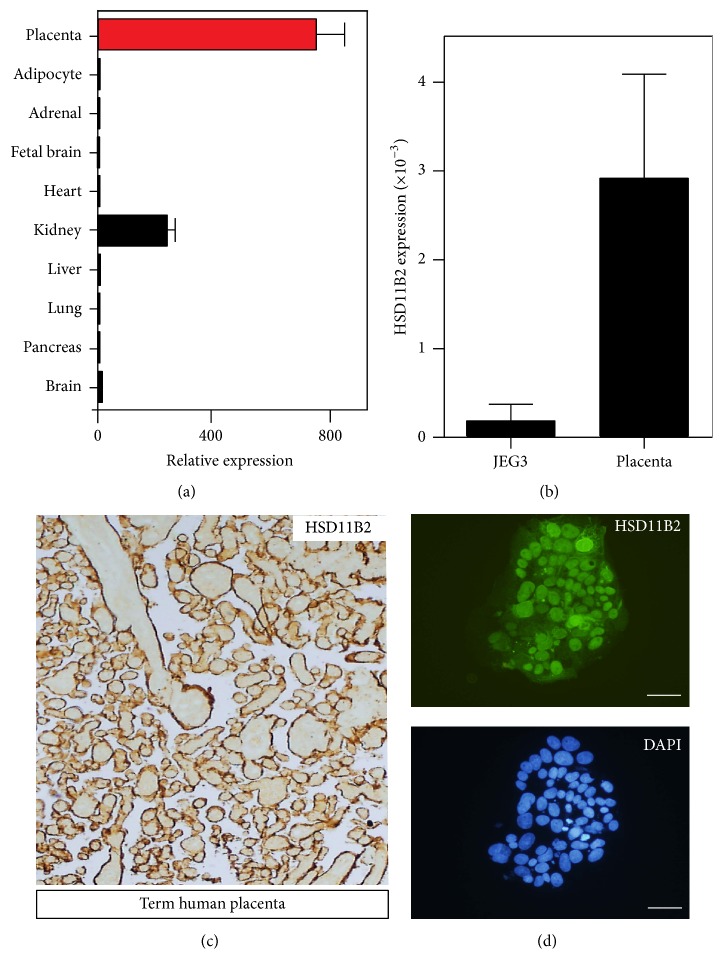
(a) Expression of data derived from the BioGPS database showing relative* HSD11B2* expression across multiple human tissues. (b) Real-time PCR showing* HSD11B2* expression in the term human placenta and in JEG-3 cells using the 2-delta-Ct method (*N* = 3, *p* > 0.05, unpaired Student's *t*-test; housekeeping gene 18S). Representative photomicrographs of (c) a term human placenta and (d) JEG-3 cells immunocytochemically stained for 11*β*-HSD2. Scale bar = 50 *μ*m.

We utilized the BioGPS database, an online platform that enables the examination of relative levels of gene expression across multiple human tissues [23]. Using this directory, we confirmed the highest levels of* HSD11B2* in the placenta, followed by the kidneys, with very little expression seen in other tissues (Figure 1(a)), which was confirmed by immunohistochemistry on human term placental samples (Figure 1(c)). We next aimed to validate the use of the human choriocarcinoma cell line, JEG-3 cells. JEG-3 cells are a widely used in vitro model of placental trophoblast cells and have previously been demonstrated to be an abundant source of endogenous 11*β*-HSD2 [24, 25]. In agreement with this, real-time PCR confirmed the expression of* HSD11B2* mRNA in JEG-3 cells, with placental RNA used as positive control (Figure 1(b)). Immunohistochemical staining preformed 24 hours after seeding also confirmed abundant expression of expression of 11*β*-HSD2 protein in JEG-3 cells (Figure 1(d)).

### 3.2. Pan-HDAC Inhibition Increases 11*β*-HSD2 Expression in JEG-3 Cells

**Figure 2:**
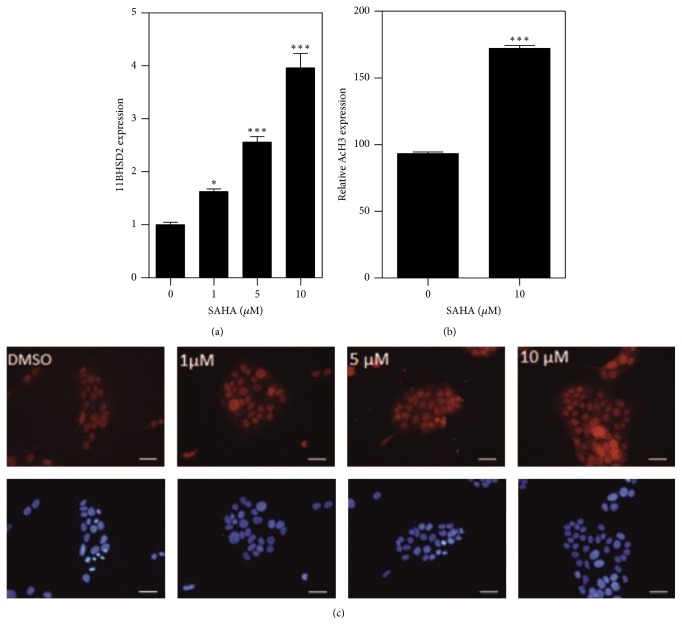
Epigenetic regulation of 11*β*-HSD2 expression. Graphical representation of (a) 11*β*-HSD2 and (b) AcH3 expression in JEG-3 cells treated with 0–10 *μ*M of SAHA for 24 h. Data are expressed as mean ± SEM. (c) Representative photomicrographs of JEG-3 cells immunocytochemically stained for 11*β*-HSD2 (^*∗*^*p* < 0.05, ^*∗∗∗*^*p* < 0.001 compared to 0 *μ*M; (a) one-way ANOVA with post hoc Tukey's and (b) unpaired Student's *t*-test; 25 cells per group per experiment; *N* = 3). Scale bar = 50 *μ*m.

**Figure 3:**
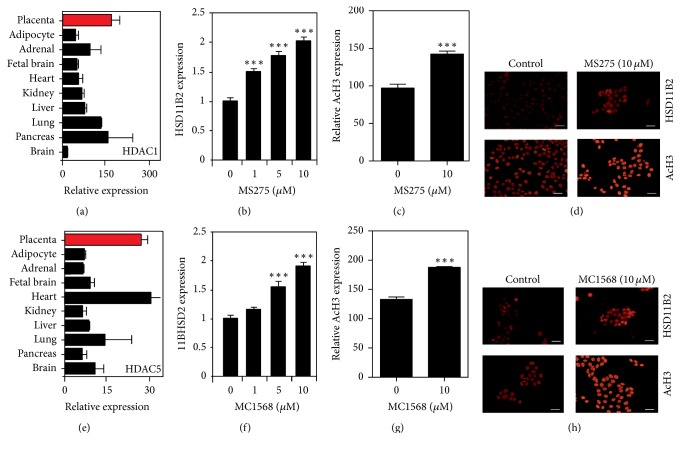
Class-specific HDACs on 11*β*-HSD2 regulation in the placenta. (a) Expression data from the BioGPS database showing the relative expression of class I HDAC, HDAC 1 in the placenta (red) relative to multiple human tissues and fetal brain. Graphical representation of (b) 11*β*-HSD2 and (c) AcH3 expression in JEG-3 cells treated with 0–10 *μ*M of class I HDAC inhibitor MS275 for 24 h. (d) Representative photomicrographs of JEG-3 cells immunocytochemically stained for 11*β*-HSD2 and AcH3 after treatment with (0–10 *μ*M) MS275 for 24 h. (e) Expression data from the BioGPS database showing the relative expression of class II HDAC, HDAC 5 in the placenta (red) relative to multiple human tissues and fetal brain. Graphical representation of (f) 11*β*-HSD2 and (g) AcH3 expression in JEG-3 cells treated with 0–10 mM of class IIa HDAC inhibitor MC1568 for 24 h. Data are expressed as mean ± SEM. (h) Representative photomicrographs of JEG-3 cells immunocytochemically stained for 11*β*-HSD2 and AcH3 after treatment with (0–10 *μ*M) MC1568 for 24 h. Data are expressed as mean ± SEM (^*∗∗∗*^*p* < 0.001 compared to 0 *μ*M; (b, f) one-way ANOVA with post hoc Tukey's and (c, g) unpaired Student's *t*-test; 25 cells per group per experiment; *N* = 3). Scale bar = 50 *μ*m.

HDACs can be divided into four distinct families, of particular interest are class I (HDAC1, HDAC2, HDAC5, and HDAC8) and class II (HDAC5, HDAC6, HDAC7, HDAC9, and HDAC10) HDACs [26]. We used the BioGPS database to examine the relative expression levels of these different HDACs in the human placenta. Class I and class II HDACs were widely expressed in the placenta (see Supplementary Figure  1 in Supplementary Material available online at https://doi.org/10.1155/2017/6169310); however, HDAC1 (class I) and HDAC5 (class IIa) had the highest relative levels of expression in the placenta compared to other tissues (Figures 3(a) and 3(e)). Given the widespread expression of HDACs, we next sought to determine the effect of global HDAC inhibition on placenta 11*β*-HSD2 protein expression. We treated JEG-3 cells with SAHA, a competitive inhibitor of both class I and class II HDACs [27]. An initial dose response experiment was carried out 24 hours after seeding where JEG-3 cells were treated with concentrations of SAHA ranging within 1–10 *μ*M for 24 h, followed by immunocytochemical staining for 11*β*-HSD2. The relative expression of 11*β*-HSD2 protein was quantified using densitometry. A one-way ANOVA revealed a significant overall effect of SAHA treatment on 11*β*-HSD2 expression (*F*_(3,8)_ = 5.5, *p* = 0.02). Tukey's post hoc test revealed a significance difference between the vehicle and 10 *μ*M SAHA group (*p* < 0.05) (Figure 2(a)). As the effects of SAHA were significant at 10 *μ*M, we also immunocytochemically stained for p-Ac-histone H3 (S11/K15) (pAcH3) in this group and found a significant increase in the levels of pAcH3 in cells treated with 10 *μ*M SAHA for 24 h (*p* < 0.001) (Figure 2(b)). Overall, these data indicate that pan-HDAC inhibition increases the levels of pAcH3 (which has been shown to correlate with gene expression) and 11*β*-HSD2 expression in JEG-3 cells.

### 3.3. Class-Specific HDAC Inhibitors (HDI) Promote 11*β*-HSD2 Expression in JEG-3 Cells

We next investigated if the effects of pan-HDAC inhibition on 11*β*-HSD2 expression were class-specific using a class I-specific HDI (MS275) [28] and a class IIa-specific HDI (MC1568) [29]. JEG-3 cells were treated with increasing concentrations (0–10 *μ*M) of MS275 or MC1568 for 24 h before being immunocytochemically stained for 11*β*-HSD2 and quantified using densitometry. A one-way ANOVA revealed a significant overall effect of both MS275 (*F*_(3,8)_ = 95.89, *p* < 0.0001) and MC1568 (*F*_(3,8)_ = 53.69, *p* < 0.0001) treatment. Tukey's post hoc test showed that MS275 or MC1568 promoted a significant increase in 11*β*-HSD2 protein expression with a significant difference observed between the control and HDI-treated groups at concentrations of 1 *μ*M (*p* < 0.05), 5 *μ*M (*p* < 0.0001), and 10 *μ*M (*p* < 0.0001) (Figures 3(b) and 3(f)). We also examined pAcH3 levels using densitometry and found a significant increase in the levels of pAcH3 in cells treated with 10 *μ*M MC1568 or MS275 for 24 h (*p* < 0.001) (Figures 3(c) and 3(g)). These data show that class I and class IIa inhibition can promote 11*β*-HSD2 protein expression in JEG-3 cells.

### 3.4. Cortisol and IL-1*β* Decrease 11*β*-HSD2 Expression Which Is Prevented by MC1568

**Figure 4:**
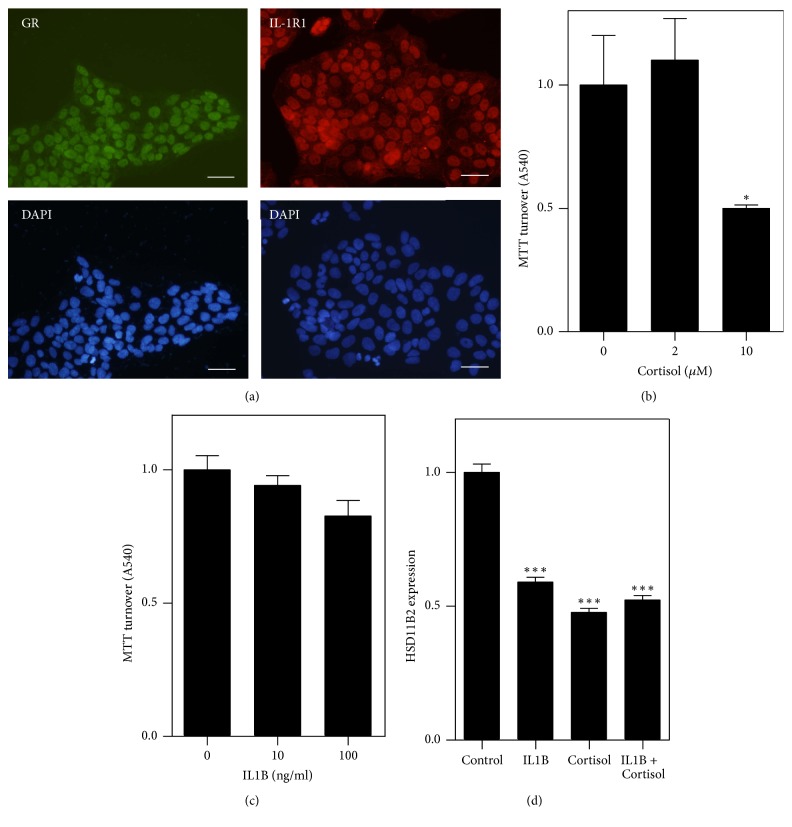
Cortisol and IL-1*β* response in JEG-3 cells. (a) Representative photomicrographs of JEG-3 cells immunocytochemically stained for the glucocorticoid receptor (GR; green) and (c) the interleukin 1 receptor, type I (IL-1R1; red). The second panel shows the corresponding DAPI stained image. (b, c) MTT assay examining the viability of JEG3 cells treated with either 0–10 *μ*M cortisol (b) or 0–100 ng/ml IL-1*β* (c) for 24 h in vitro. (d) Graphical representation showing the levels 11*β*-HSD2 in JEG-3 cells exposed to a vehicle (control), 10 ng/ml IL-1*β* or 2 *μ*M cortisol for 24 h. Data are expressed as mean ± SEM (^*∗*^*p* < 0.05 compared to 0 *μ*M, ^*∗∗∗*^*p* < 0.001 compared to control; one-way ANOVA with post hoc Tukey's; (d) 100 cells per group per experiment; *N* = 3). Scale bar = 50 *μ*m.

Given that alterations in placental* HSD11B2* expression are seen in pregnancies complicated with stress or infection [5, 7], we next sought to determine if the biological mediators of stress (Cort) and infection (IL-1*β*) altered 11*β*-HSD2 protein expression at the cellular level. Having confirmed using immunocytochemistry that the glucocorticoid receptor (GR) and interleukin 1 receptor, type I (IL1R1), were expressed in JEG-3 cells (Figure 4(a)), we carried out an MTT assay to establish a concentration of Cort and IL-1*β* that did not affect cell viability. JEG-3 cells were treated with Cort (0–10 *μ*M) or IL-1*β* (0–100 ng/ml) for 24 h and MTT assays were performed. An ANOVA showed an overall effect of Cort and IL-1*β* treatment on cell viability, with a difference observed with 10 *μ*M Cort (Figure 4(b)) and 100 ng/ml IL-1*β* (Figure 4(c)) groups (*p* < 0.05). JEG-3 cells were then treated with 2 *μ*M of Cort or 10 ng/ml IL-1*β* (concentrations that did not affect cell viability) for 24 h before being fixed and immunocytochemically stained for 11*β*-HSD2. Using densitometry, we observed a reduction in 11*β*-HSD2 protein expression following exposure to Cort and IL-1*β* (Figure 4(d)).

### 3.5. HDIs Can Restore 11*β*-HSD2 Expression in an Environment of Stress and Inflammation

**Figure 5:**
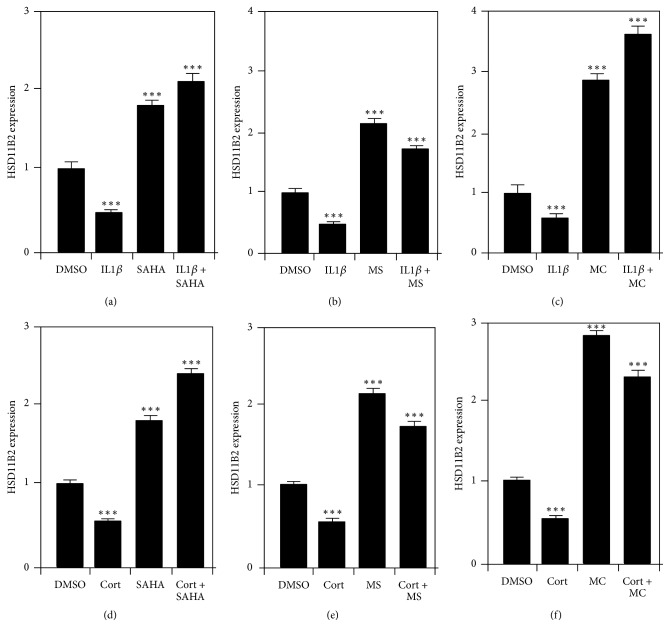
SAHA, MS275, and MC1568 prevent cortisol and IL1*β*-induced decreases in 11*β*-HSD2 expression. Graphical representation and 11*β*-HSD2 expression in JEG-3 cells treated with 2 *μ*M Cort or 10 ng/ml IL-1*β* in the presence or absence of 10 *μ*M SAHA (a, d), MS275 (b, e), or MC1568 (c, f) for 24 h. Data are expressed as mean ± SEM. (^*∗∗∗*^*p* < 0.001 compared to DMSO; one-way ANOVA with post hoc Tukey's test; 25 cells per group per experiment; *N* = 3.)

After identifying Cort and IL-1*β* as potential biological mediators causing a decrease in 11*β*-HSD2 protein expression, we next aimed to determine if HDIs could counteract these effects of cortisol and IL-1*β* on 11*β*-HSD2 protein expression. After plating for 24 hours, JEG-3 cells were treated with 10 *μ*M of SAHA, MC1568, or MS275 followed by cortisol or IL-1*β* before being fixed and immunocytochemically stained for 11*β*-HSD2 protein. Densitometry revealed that pretreatment of JEG-3 cells with nonspecific inhibitor SAHA attenuated the effect of IL-1*β* and Cort (SAHA: 2.8 ± 0.15; SAHA + IL-1*β*: 3.0 ± 0.2; SAHA + Cort: 3.350 ± 0.19) (Figures 5(a) and 5(d)) on 11*β*-HSD2 expression. Similarly, treatment of JEG-3 cells with either class I-specific HDI, MS275 (MS275: 2.2 ± 0.06; MS275 + IL-1*β*: 3.18 ± 0.06; MS275 + Cort: 2.2 ± 0.09) (Figures 5(b) and 5(e)), or class IIa-specific HDI MC1568 (MC1568: 1.8 ± 0.08; MC1568 + IL-1*β*: 2.3 ± 0.1; MC1568 + Cort: 1.4 ± 0.07) (Figures 5(c) and 5(f)) was sufficient to attenuate the effect of both Cort and IL-1*β* on 11*β*-HSD2 expression. These data show that exposure to heightened levels of Cort and IL-1*β* can reduce the levels of 11*β*-HSD2 protein in JEG-3 cells and that this effect that can be prevented by HDAC inhibition.

## 4. Discussion

The aim of this study was to examine the role of epigenetic regulators in the control of 11*β*-HSD2 protein expression in placental cells. We used the in vitro placental model JEG-3 cells, as, despite their limitations, they are a well-established cell line commonly used to mimic placental trophoblast cells [30]. We employed pharmacological inhibitors of HDACs to modulate histone acetylation and examined the impact of this on 11*β*-HSD2 protein expression. Finally, to assess the potential of these compounds to regulate 11*β*-HSD2 expression under conditions of stress and inflammation, cells were exposed to biological mediators of these conditions, namely, exogenous cortisol and IL-1*β*.

*HSD11B2* has previously been shown to localise in trophoblast cells, with highest expression observed in the syncytiotrophoblast [31, 32]. In line with these studies, we demonstrated that 11*β*-HSD2 protein is strongly expressed in the term human placenta. To model trophoblast cells in vitro, we used the human choriocarcinoma cell line, JEG-3 cells. We found that these cells express* HSD11B2* mRNA making them a useful and convenient model to examine the molecular mechanisms that regulate 11*β*-HSD2 expression.

Using the BioGPS database, we demonstrated high expression of class 1 HDACs 1, 2, 3, and 8 and class 2 HDACs 5, 4, 7, and 9, suggesting a role for HDAC proteins in the placenta. Based on these findings, we used a SAHA, a pan-HDAC inhibitor, and demonstrated a dose-dependent increase in 11*β*-HSD2 protein expression. To confirm that the increase in 11*β*-HSD2 protein expression was paralleled by an increase in histone acetylation, we immunocytochemically stained the cells for AcH3 and showed a similar dose-dependent increase AcH3. This is in contradiction to previous studies, where* HSD11B2* expression was reported to be unchanged in JEG-3 cells following treatment with broad-spectrum class I and class II inhibitor trichostatin A [24]. However, the dose of TSA (300 nm) used in these studies was much smaller than the dose at which we observed an effect (10 uM) and we have identified that the effect of HDAC inhibition on 11*β*-HSD2 expression is dose-dependent.

HDACs play a diverse role during fetal development [26]. Global knockdown of HDAC3 [33] HDAC1 [34] and HDAC7 [34] results in fetal lethality; however, mice lacking HDAC6 develop normally [35]. HDACs have also been shown to be important regulators of placental development as inhibition of class II HDACs has been shown to impair trophoblast differentiation through interactions with Hypoxia-inducible factor [36]. Additionally, interaction of HDACs with the STAT-1 TF may contribute to inhibition of IFN-*γ*-inducible gene expression in trophoblast cells, thereby protecting the placenta cells from maternal immune rejection and contributing to a successful pregnancy [37]. This broad range of functions of HDACs suggests that global inhibition could result in detrimental effects; therefore, a more specific inhibition could represent an optimal method for modifying 11*β*-HSD2 expression. To determine if HDAC regulation of 11*β*-HSD2 protein expression is class-specific, we used class-specific pharmacological HDAC inhibitors. We observed a similar increase in 11*β*-HSD2 protein expression with class-specific inhibition of either class I or class IIa HDACs, suggesting that many HDACS are likely involved in regulating 11*β*-HSD2 protein expression. Whilst this is the first study to examine the effects of class-specific inhibitors on HSD11B2 expression, it is interesting to note that previous studies have demonstrated a class-specific effect of HDACs on the regulation of other placental genes. Specifically, matrix metalloproteinase 9 has been shown to be regulated by class II but not class I HDACs [38].

Placental* HSD11B2* has been shown to be reduced in a number of adverse pregnancy conditions including anxiety, stress, and infection [5–7]. As elevations in proinflammatory cytokines and steroids are observed in these conditions [39, 40], we used cortisol and IL-1*β* to mimic an environment of stress and inflammation. We have previously demonstrated a reduction in 11*β*-HSD2 protein expression in JEG-3 cells following administration of IL-1*β* [7]. In this study, we also report a decrease in 11*β*-HSD2 expression following cortisol administration. In contrast, Ni and colleagues have previously shown an increase in 11*β*-HSD2 expression in primary human trophoblast cells exposed to Cort [17]. However, this study used primary cells which highlights the need for further study of these questions in primary trophoblast cells. Additionally, the maximum dose of cortisol used was 1 *μ*M, whereby we observed a decrease at 2 *μ*M. It is possible that cortisol may act in an adaptive way to induce 11*β*-HSD2, thereby protecting the fetus from high maternal glucocorticoids but, at a certain threshold cortisol, may begin to negatively impact 11*β*-HSD2 expression. Interestingly, broad or either class-specific HDAC inhibitors were sufficient to prevent the cortisol and IL-1*β*-induced decreases in 11*β*-HSD2 expression. This raises the possibility of targeting key epigenetics modulators to protect the fetal glucocorticoid barrier and untimely fetal glucocorticoid overexposure. However, given the critical role of epigenetic marks in fetal development, nonspecific inhibition of HDACs, even at class level, could produce detrimental effects on fetal development; therefore, identifying more specifically the precise epigenetic mechanism mediating HSD11B2 regulation using knockdown or overexpression of individual HDACs would allow the development of a more targeted approach. The advancement of targeted nanoparticles to deliver chemotherapeutic agents directly to the placenta represents an exciting new avenue to alter placental epigenetic mediators without interfering with the fetus [41]. Notably, we also observe potentiation of the effects of SAHA on HSD11B2 expression when administered with cortisol. Once activated, the GR can bind to many coactivator proteins with known HAT activity [42]. The combined inhibition of HDACs by SAHA with the potential increase in HAT activity caused by GR activation from exogenous cortisol may explain this enhanced 11*β*-HSD2 protein expression. This relationship further highlights the complexity of 11*β*-HSD2 regulation and the epigenetic landscape and confirms the need for more studies examining how placental 11*β*-HSD2 protein is controlled under both basal and pathological conditions.

Here, we provide evidence of a role for histone acetylation in the regulation of 11*β*-HSD2 in the placenta; a limitation is that the present study used JEG-3 cells. Although we confirmed 11*β*-HSD2 to be abundantly expressed in this cell line and that* HSD11B2* levels are comparable between JEG-3 cells and the human placenta, there are potential caveats associated with using JEG-3 cells [43]. As such replicating the current study in primary trophoblasts will help to clarify the functional role of HDACs in the regulation of 11*β*-HSD2 protein expression in the placenta. However, the present study demonstrates a role for HDACs in the regulation of a key enzyme that maintains the fetal glucocorticoid barrier under basal and pathological conditions. It is likely that a combination of different epigenetic modifiers including HDACs are involved in regulating 11*β*-HSD2 expression. As HDACs have a broad role in regulating fetal development, inhibition of all HDACs could be detrimental to the developing fetus. Therefore, unravelling the role of individual HDACs in 11*β*-HSD2 regulation, using more specific pharmacological inhibitors or targeted knockdown of HDACs, will be crucial to understanding the epigenetic mechanisms that regulate 11*β*-HSD2 expression and for developing novel protective pharmacotherapies for the human placenta.

## Supplementary Material

Relative expression levels of Class I and Class II HDACs in the placenta (red) compared to other human tissues assessed using the BioGPS database. Both Class I and Class II HDACs are abundantly expressed in the human placenta.
