# Supplementary material for: Class-Specific Histone Deacetylase Inhibitors Promote 11-Beta Hydroxysteroid Dehydrogenase Type 2 Expression in JEG-3 Cells
Source: Int J Cell Biol. 2017 Feb 21;2017:6169310. doi: 10.1155/2017/6169310 (PMC5339487; doi:10.1155/2017/6169310)
Supplement: Supplementary file 1 — Relative expression levels of Class I and Class II HDACs in the placenta (red) compared to other human tissues assessed using the BioGPS database. Both Class I and Class II HDACs are abundantly expressed in the human placenta. [file 6169310.f1.pdf]

Supplementary Figures:

Supplementary Figure 1:

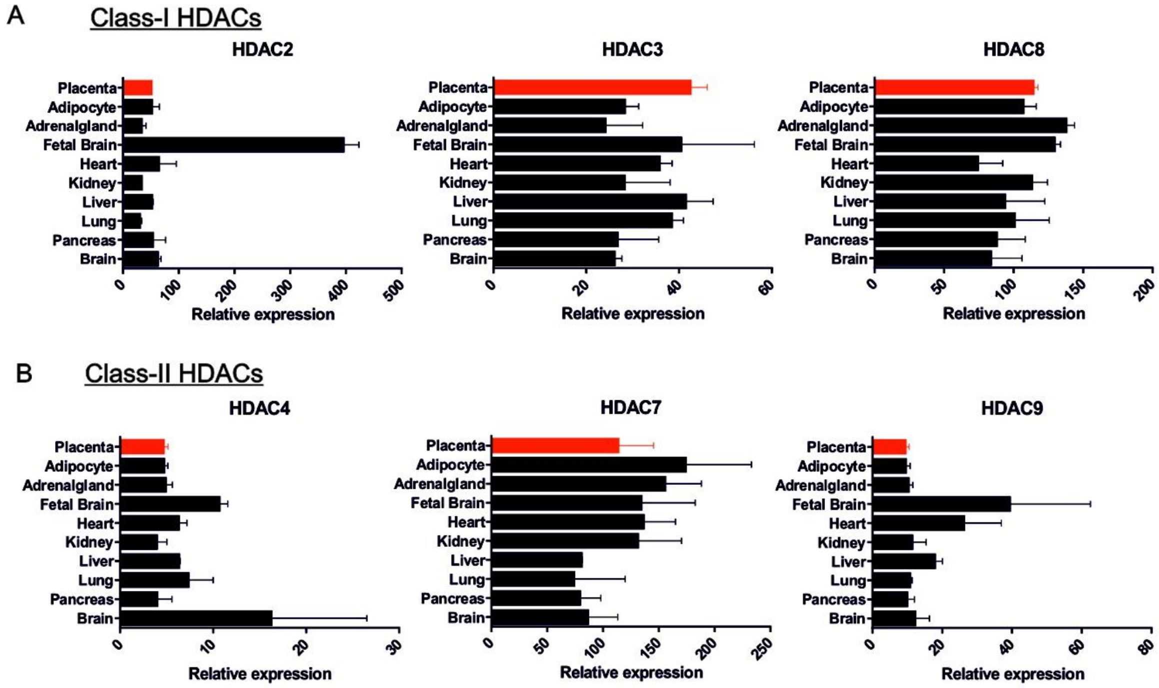

Supplementary Figure 1: Expression levels of different classes of HDACs: (A,B) Expression data from the BioGPS database showing the relative expression of (A) Class-I HDACs, HDAC 2, 3 and 8 and (B) Class-I Ia HDACs, HDAC 4, 7 and 9 in the placenta (red) relative to multiple human tissues and fetal brain.
